# Intraoperative Iatrogenic Aortic Dissection in Cardiovascular Surgery: Case Series and Literature Review

**DOI:** 10.3390/jcdd13010005

**Published:** 2025-12-21

**Authors:** Jinjing Wu, Tiantian Sun, Peirong Lin, Sheng Wang

**Affiliations:** Department of Anesthesiology, Beijing Anzhen Hospital, Capital Medical University-Beijing Institute of Heart Lung and Blood Vessel Diseases, Beijing 100029, China; grejiayou@163.com (J.W.); stiann00@163.com (T.S.)

**Keywords:** iatrogenic aortic dissection, cardiac surgery, intraoperative complications, mortality

## Abstract

Background: Intraoperative iatrogenic aortic dissection (IAD) is an uncommon but serious complication of cardiac surgery, and available evidence remains limited, with most reports based on small series. This study summarizes our experience in a high-volume cardiovascular center and compares the findings with published data. Methods: We retrospectively reviewed 31 consecutive IAD cases treated at Anzhen Hospital from 2020 to 2024, assessing patient characteristics, operative details, and postoperative outcomes. Results: IAD was identified intraoperatively in 90.3% of patients, with ascending aortic involvement in 80.6%. The main procedures included ascending aorta replacement (45.2%) and hemiarch replacement (22.6%). Mean cardiopulmonary bypass time was 342.6 ± 133.8 min, and 38.7% required circulatory arrest. Major postoperative complications were low cardiac output syndrome (61.3%), neurological injury (25.8%), and acute kidney injury (45.2%). Overall mortality was 38.7%. Review of 17 original studies (1998–2025; >2000 patients) showed a pooled mortality of 32.8%. Patients in our cohort had higher operative risk and more complex procedures, which may partly explain the higher complication and mortality rates. Conclusions: IAD remains a major intraoperative challenge. Prompt recognition and individualized surgical strategies are essential. These findings provide further insight into intraoperative iatrogenic dissection and may help inform operative and perioperative decision-making.

## 1. Introduction

Intraoperative iatrogenic aortic dissection (IAD) is rarely seen in cardiac operations but can lead to catastrophic consequences once it occurs. Reported incidence rates vary considerably, ranging from 0.06% to 0.23% [1,2], depending on the type of surgery performed and the method of aortic cannulation. Data collected from the Society of Thoracic Surgeons (STS) registry, which included over two million cardiac procedures, found an incidence of only 0.058%, but almost half of those patients did not survive [3,4].

Despite the long-standing awareness of this complication, most reports on IAD remain small, retrospective, and single-center [5,6]. The lack of standardized reporting and differences in operative practice make it difficult to summarize the overall risk or establish clear recommendations for prevention and management. Direct comparisons between centers are also rarely available [7].

To address this knowledge gap, we reviewed 31 consecutive IAD cases treated at Anzhen Hospital, a high-volume cardiac surgery center performing more than 23,000 procedures annually with an operative mortality of 0.37%. These figures refer exclusively to surgical operations and do not include catheter-based or interventional cardiology procedures. In addition to describing our experience, we compared our findings with 17 published studies (>2000 patients, 1998–2025) to better understand how our outcomes align with or differ from previously reported results.

## 2. Methods

### 2.1. Study Design

We retrospectively reviewed patients undergoing cardiac surgery at Anzhen Hospital, Beijing, China. Ultimately, 31 patients suffered from IAD from May 2020 to December 2024. This study was approved by the hospital’s Institutional Review Board (NO: 2025025x). Informed consent was waived as the study involved a review of existing medical records.

This retrospective analysis was to uncover the incidence of IAD, its management, and the outcomes at our center. To place our findings in context, we also reviewed 17 previously published studies (1998–2025). From these reports, we collected information such as patient age, duration of cardiopulmonary bypass, and rates of neurological injury, renal failure, and other complications, and compared these with our own results to show how management and outcomes may differ between centers.

### 2.2. Patient Population and Data Collection

During the study period, 31 patients who developed IAD during or immediately after cardiac surgery were identified using the hospital’s electronic medical records and operative databases. Data collection followed definitions used in the STS Adult Cardiac Surgery Database and other large registries.

Patients were included if they had a new aortic dissection detected either intraoperatively with transesophageal echocardiography (TEE) or postoperatively on computed tomography (CT) or aortography, and if the dissection occurred during surgery or within 7 days after the procedure. Patients with known preoperative dissection, chronic dissection, or trauma-related dissections were not included. Iatrogenic dissections were categorized according to the TEM (type–entry–malperfusion) system. Type classification was based on whether the ascending aorta was involved. The entry was defined as the anatomical location of the intimal disruption as identified intraoperatively. Because the events occurred under cardiopulmonary bypass and general anesthesia, clinical manifestations of malperfusion could not be assessed. Preoperative coronary malperfusion was defined as objective evidence of impaired myocardial perfusion based on angiography, coronary CT, or ischemic ECG/biomarker findings.

### 2.3. Surgical Management

If IAD occurred before CPB, arterial pressure was lowered, and CPB was initiated via the axillary or femoral artery, with TEE used to confirm true-lumen perfusion. The ascending aorta was then exposed and repaired under hypothermia. If IAD occurred after CPB had been initiated through an aortic cannula, flow through the original cannula was reduced, and arterial inflow was redirected to a peripheral artery under TEE guidance while CPB was maintained. Hypothermic aortic repair was then performed. For cerebral protection, selective cerebral perfusion combined with moderate systemic cooling (25–28 °C) was used. Because intraoperative IAD occurred during ongoing cardiac surgery, root intervention—when required—was incorporated into composite procedures rather than performed as isolated Bentall or valve-sparing (David) operations. Root management, therefore, consisted mainly of aortic root/ascending aorta replacement, either alone or in combination with hemiarch replacement, total-arch replacement with a stented elephant trunk (Sun’s procedure), or other concomitant repairs dictated by the primary cardiac operation.

### 2.4. Outcome Measures

The main outcome was in-hospital mortality. Secondary outcomes included neurological complications (stroke and spinal cord injury), cardiovascular events (low cardiac output syndrome, heart failure, atrial or ventricular arrhythmias), dysfunction of the liver or kidneys, respiratory failure, bleeding or clotting problems, and infections. The definitions for these clinical events followed previously published standards [8].

### 2.5. Statistical Analysis

All statistical analysis was conducted using SPSS software (version 22.0; IBM Corp., Armonk, NY, USA). The Shapiro–Wilk test was completed for normal distribution verification of continuous data. Results were accordingly reported as mean with standard deviation or as median with interquartile range. Categorical data are shown as counts and percentages. Results from previous literature are only summarized descriptively due to the diverse study designs and outcomes. No pooled meta-analysis was attempted.

## 3. Results

### 3.1. Demographics and Clinical Presentation

The average age in our cohort was 65 years, and 48.4% of them were women. BMI values suggested a normal weight range. Preoperative echocardiography showed that left ventricular systolic function was generally preserved; only 2 of 31 had a marked reduction of EF < 30%. Some patients had preexisting structural aortic abnormalities, including dilated ascending aorta and bicuspid valve (16.1%) and aortic aneurysm (9.7%). Hypertension was very common (67.7%), and advanced heart failure was frequently present (16.1%). Other associated conditions included atrial fibrillation (16.1%), preoperative dialysis (3.2%), and diabetes (12.9%). Several patients had undergone cardiac surgery in the past (22.6%), and coronary involvement was also common, with many experiencing stable angina (38.7%) or signs of malperfusion (48.4%). Detailed information is summarized in Table 1.

### 3.2. Surgical Characteristics and Intraoperative Findings

Minimally invasive mitral valve repair or replacement accounted for roughly one-fifth of surgeries (22.6%), followed by isolated coronary artery bypass grafting (CABG, 12.9%) and isolated aortic valve surgery (9.7%). Combined surgery contributed to the largest proportion (32.3%), involving double-valve repair or CABG together with valve or ventricular repair. Only a few of the cases occurred in transcatheter aortic valve replacement (TAVR) (6.5%) and isolated aortic procedures (6.5%), and all TAVR-related dissections were recognized intraoperatively.

Most dissections were recognized during surgery (90.3%), highlighting the value of continuous intraoperative monitoring. Surgeons relied primarily on direct visual inspection of the aorta (51.6%) and transesophageal echocardiography (TEE, 38.7%) for diagnosis. Postoperative imaging—computed tomography or aortography—was carried out postoperatively in rare situations (each 3.2%), which further supports the reliability of intraoperative detection before major hemodynamic problems developed. Using the TEM framework, 30 patients (96.8%) were classified as Type A dissections involving the ascending aorta, whereas one patient (3.2%) with an entry tear in the descending thoracic aorta was classified as Type B. The entry tear was most frequently located on the ascending aorta (80.6%), followed by aortic cannulation sites (9.7%), clamp-associated entry (6.5%), and the descending aorta (3.2%). Among intraoperative procedural triggers, arterial cannulation, cardioplegia cannulation, and aortic clamping were implicated in approximately one-quarter of cases each (25.8%), consistent with previous studies that describe these maneuvers as mechanically stressful. Other manipulation accounted for 9.7%, and no identifiable trigger could be detected in 12.9% of patients (Table 2).

These observations highlight the technical challenges of aortic manipulation during cardiac procedures. Cannulation and clamping steps remain particularly critical for preventing dissection. Empirical intraoperative findings and TEE continue to play a major role in timely detection and intervention, which is essential for better future outcomes.

### 3.3. Operative and Intraoperative Characteristics

Different cannulation techniques were applied due to differences in patients and surgeries. The most common setups included vena cava–aortic (29.0%) and right atrium–femoral (25.8%). Internal jugular vein–femoral access was chosen in 19.4% of cases. Less frequent options were femoral–femoral and right atrium–aortic cannulation. Surgeons selected the approach according to vessel condition, hemodynamics, and the anticipated need for circulatory support.

The mean operative time was 10 ± 22.9 h, reflecting the combined duration of the index cardiac procedure and the subsequent repair of the intraoperative aortic dissection. Most procedures required reconstruction of the aortic root or ascending aorta (45.2%). In 22.6% of patients, this was combined with hemiarch replacement, reflecting more extensive dissection. A smaller group underwent Sun’s procedure (12.9%), while 6.4% of patients had hybrid operations that combined open aortic repair with endovascular techniques (EVAR or TEVAR). Ascending aortoplasty was used in 9.7% of cases, generally for localized disease not requiring complete root replacement. On-pump management involved long cardiopulmonary bypass (CPB) times, with an average of 342.6 ± 133.8 min. The cross-clamp was kept in place for a mean of 165 ± 70.4 min. Total circulatory arrest was necessary in 38.7% of operations, lasting on average 28.7 ± 19.3 min. In 19.4% of cases, only the lower body was arrested, for an average of 32.3 ± 9.7 min. Standard hypothermic protocols were followed. The average nasopharyngeal and rectal temperature was 25.9 °C and 26.2, respectively (Table 3).

These numbers show the technical difficulty of treating iatrogenic aortic dissection during cardiac surgery. Many patients needed circulatory arrest, had long bypass durations, and underwent complex reconstructions. Surgeons often performed distinct cannulation and repair strategies, highlighting the need for patient-specific operative planning and careful perfusion management. Continuous intraoperative monitoring was essential for maintaining safety and improving outcomes.

### 3.4. Postoperative Outcomes and Complications

The average intraoperative red blood cell transfusion volume was 9.5 ± 5.3 units. Patients had a median hospital stay of 18 days, reflecting the prolonged recovery associated with IAD. A substantial proportion of patients (29%) required reoperation for bleeding control or other complications, while 6.5% required ECMO support as rescue therapy for refractory cardiocirculatory failure, including failure to wean from CPB or refractory cardiogenic shock. Neurological complications were reported in 25.8% of patients, with stroke (22.6%) and permanent spinal cord injury (3.2%). Cardiovascular complications were the most prevalent, affecting 61.3% of cases. Heart failure was observed in 35.5%, while atrial fibrillation occurred in 12.9%. These findings indicate a substantial burden of postoperative cardiac dysfunction, necessitating close hemodynamic monitoring and management. Hepatic and renal dysfunction were significant postoperative concerns, with renal failure affecting 45.2% of cases and hepatic dysfunction observed in 16.1%. These complications likely reflect the systemic impact of prolonged cardiopulmonary bypass and circulatory arrest, further underscoring the importance of perioperative organ protection strategies.

Respiratory complications were present in 58.1% of cases, including pneumonia (16.1%), tracheostomy (16.1%), and pleural effusion (12.9%), often requiring prolonged ventilatory support. Hemorrhagic and thrombotic events occurred in 16.1% of cases, with re-exploration for bleeding required in 6.5% of patients, and deep vein thrombosis (6.5%) or arteriovenous fistula formation (3.2%). Infectious complications developed in 12.9% of patients, with sepsis and gastrointestinal infections each affecting 6.5%. These infections likely contributed to increased morbidity and prolonged hospitalization.

In-hospital mortality was 29%, and 9.7% of patients died within 30 days postoperatively. Cumulatively, 38.7% of the cohort died during the follow-up period, reflecting the substantial long-term mortality burden in this population (Table 4).

## 4. Discussion

In this single-center series of 31 patients with intraoperative iatrogenic aortic dissection (IAD), most events were recognized and managed during the index operation, consistent with contemporary reports in which the vast majority of iatrogenic type A dissections are diagnosed intraoperatively or in the immediate postoperative period. Our overall 30-day mortality of 38.7% lies at the upper end of previously reported ranges (approximately 20–45%) and is similar to the early mortality observed in recent large multi-center and single-center series of iatrogenic type A dissection. Compared with the largest contemporary cohorts by von Aspern et al. (92 patients, 44.6% early mortality) and Biancari et al. (103 patients, 30.1% early mortality) [9,10], our outcomes appear broadly comparable despite differences in case mix and surgical strategies, underscoring the persistently high lethality of IAD across centers (Table 5). Neurological complications and renal dysfunction were also observed more frequently than in several earlier series, which may reflect our higher proportion of complex and redo procedures, as well as more systematic detection and reporting of organ dysfunction [9].

While these differences may be driven in part by patient complexity and operative strategy rather than perioperative care alone, they highlight that once IAD occurs, outcomes depend largely on the interplay between rapid recognition, accurate anatomical assessment, and an appropriate reconstruction plan rather than the choice of a single superior operative technique. In interpreting our findings, it is important to consider the effect of cardiopulmonary bypass (CPB) duration. The CPB times in our cohort were longer than those reported in most previous IAD series, in which median or mean perfusion durations typically ranged between 180 and 230 min, whereas our mean time exceeded 340 min. This reflects a larger proportion of patients undergoing combined or redo procedures and our institutional preference to complete aortic and coronary reconstructions in a single session when feasible. Prolonged CPB is a well-recognized contributor to postoperative complications, and the extended perfusion times in our cohort likely played an important role in the higher rates of renal, respiratory, and neurological events compared with some other series. Longer perfusion increases inflammatory stress and reduces organ perfusion, which may further compound the physiological burden created by intraoperative dissection and extensive aortic repair. These factors together provide a plausible explanation for the complication pattern observed in our patients [11].

In addition to these operative factors, intrinsic aortic wall characteristics may also be relevant. In our cohort, 5 of the 31 patients (16.1%) had a bicuspid aortic valve (BAV), compared with an estimated prevalence of 1–2% in the general population. This overrepresentation is consistent with the recognized association between BAV and ascending aortic medial degeneration and aneurysmal disease, which may confer increased vulnerability of the aortic wall during operative manipulation. While most existing research has focused on spontaneous acute type A dissection in the context of BAV-associated aortopathy, current guidelines and observational data acknowledge BAV-related aortic pathology as a risk factor for dissection but provide little specific guidance on its role in intraoperative iatrogenic events. Our findings therefore suggest a possible increased susceptibility to IAD in BAV patients, although the true magnitude and clinical relevance of this association will need to be clarified in future prospective studies. Comparison with published series further highlights the pronounced heterogeneity across centers, driven by variations in baseline risk profiles, mechanisms of injury, perfusion, and cerebral protection protocols, and the extent of aortic reconstruction. For example, von Aspern et al. [9] reported a higher proportion of isolated ascending aortic replacement, whereas other cohorts included more extensive arch procedures and fewer redo or combined operations, which likely contributed to shorter CPB times and different complication profiles than observed in our series. In addition, inconsistencies in endpoint definitions and observation windows complicate cross-study comparisons: while we included all deaths within 30 postoperative days, several earlier reports counted only intraoperative or in-hospital deaths, which may underestimate early mortality and partly explain the lower rates reported in some series. These discrepancies emphasize the need for standardized definitions and core outcome sets in future collaborative studies.

**Table 5:** Comparison of selected perioperative parameters and major outcomes of IAD cases between our center and published series.

Study	Year	*n*	Age (yrs)	CPB (min)	Neuro Comp	Renal Comp	ECMO	Mortality
Our center	2025	31	65.0 ± 7.2	342.6 ± 133.8	8/31 (25.8%)	14/31 (45.2%)	2/31 (6.5%)	12/31 (38.7%)
Ruchat et al. [12]	1998	7	64 (62–69.5)	NA	NA	NA	NA	4/10 (40%)
Januzzi et al. [13]	2002	34	71.4 ± 9.4	NA	NA	NA	NA	12/34 (35%)
Fleck et al. [14]	2006	7	69.2 ± 7.6	192 (median)	NA	NA	1/7 (14.3%)	3/7 (43%)
Ketenci et al. [15]	2008	21	62.1 ± 8	NA	7/21 (33.3%)	2/21 (9.5%)	NA	10/21 (47.6%)
Hurt et al. [16]	2008	33	NA	147 ± 77	NA	7/33 (21.2%)	NA	8/33 (24.2%)
Lin et al. [17]	2009	7	70.3 ± 8.4	NA	1/7 (14.3%)	1/7 (14.3%)	0/7 (0%)	3/7 (42.9%)
Hwang et al. [18]	2010	10	62.4 ± 8	NA	1/10 (10%)	NA	0/10 (0%)	4/10 (40%)
Williams et al. [1]	2010	1294	72 (64–77)	NA	119/1294 (9.2%)	175/1294 (13.5%)	NA	615/1294 (47.5%)
Leontyev et al. [19]	2012	48	66 ± 14	222 ± 93	6/48 (12.5%)	15/48 (31.3%)	NA	20/48 (41.7%)
Rylski et al. [20]	2013	100	67.7 ± 9.4	188 (IQR 134–230)	4/100 (4.0%)	1/100 (1.0%)	NA	16/100 (16.0%)
Narayan et al. [21]	2015	7	72 (69–75)	193 (range 120–262)	0/7 (0%)	5/7 (71.4%)	NA	2/7 (28.6%)
Shea et al. [22]	2019	15	74.0 ± 7.3	229.5 ± 212.7	1/15 (6.7%)	4/15 (26.7%)	1/15 (6.7%)	1/15 (6.7%)
von Aspern et al. [9]	2022	92	68.6 ± 12	205 ± 80	16/92 (17.4%)	32/92 (34.8%)	NA	41/92 (44.6%)
Wang et al. [11]	2022	21	65.0 (56.5–73)	217.5 (IQR 156–257)	4/21 (19.0%)	5/21 (23.8%)	NA	2/21 (9.5%)
Biancari et al. [10]	2022	103	69.2 ± 9.4	NA	22/103 (21.4%)	16/103 (15.5%)	9/103 (8.7%)	31/103 (30.1%)
Harris et al. [23]	2025	333	68.7 (weighted mean)	NA	4/221 (1.8%)	19/213 (8.9%)	NA	73/329 (22.2%)
Bauer et al. [24]	2025	8	73 (IQR 70–75)	257 (IQR 208–336)	1/8 (12.5%)	2/8 (25%)	2/8 (25%)	3/8 (37.5)

Variables are presented as *n* (%), mean ± standard deviation, or median (IQR).

Although the majority of cases in our series involved intraoperative IAD during open cardiac surgery, two events occurred in the setting of TAVR, and similar events have been reported during catheter-based interventions. A recent analysis from the STS Adult Cardiac Surgery Database (2017–2023) found that iatrogenic acute type A dissections during catheter-based procedures carried a significantly higher operative mortality than spontaneous cases (31.9% vs. 18.2%), with TAVR and PCI being the most frequent precipitating procedures and TAVR showing the highest mortality (43.0%) among them. These findings underscore that, irrespective of the procedural approach, the combination of procedural trauma and underlying aortic pathology can result in aortic dissection with substantial mortality risk, highlighting the need for heightened vigilance in high-risk patient populations [25].

Limited exposure and suboptimal instrument angles in minimally invasive procedures may exacerbate this risk. Once peripheral-cannulation-related IAD is suspected, prompt cessation of peripheral CPB, expedited conversion to sternotomy, and early establishment of antegrade central perfusion are considered key steps to prevent ongoing false-lumen pressurization and limit malperfusion-related injury. In our series, MICS ranked second only to combined procedures as the most frequent operative setting for IAD. A recent single-center study reported 8 cases of IAD during MICS, representing an incidence of 0.75% among 1070 procedures—comparable to or exceeding that in some full-sternotomy CABG or valve surgery series. All patients required conversion to sternotomy for repair, and the in-hospital mortality was 37.5%, similar to our cohort (38.7%). The authors emphasized that incomplete preoperative aortic imaging, heavy calcification, and challenging peripheral access increased the risk, underscoring the need for comprehensive preoperative assessment when side-clamping or alternative approaches are planned. Early intraoperative warning signs included abrupt right radial arterial pressure drops and cerebral oximetry (cNIRS) decline, supporting the use of multi-parametric monitoring (invasive arterial pressure, cerebral oximetry, and TEE) in high-risk patients to shorten recognition delays. These findings, together with our data, suggest that once IAD occurs, prognosis is determined less by the surgical access route than by the speed of diagnosis and adequacy of reconstruction [24]. Concerns that the expansion of off-pump coronary artery bypass (OPCAB) surgery could increase IAD incidence remain unresolved; our findings support the view that the critical determinant is the quality of aortic handling in the context of individual anatomy and pathology. For OPCAB cases requiring side clamping, preoperative imaging should identify calcified or fragile segments, with consideration given to clamp-free techniques, proximal anastomotic devices, or early conversion to cardiopulmonary bypass [16].

The main strengths of this study include the use of consecutive cases from a high-volume cardiac surgery center, providing real-world insight into IAD management, and the incorporation of a structured literature comparison to contextualize our findings. It should also be noted that established ATAAD risk scores such as GERAADA and ERTAAD are not applicable to intraoperative IAD, because the key symptom- and presentation-based variables required for score calculation (such as pain, pre-presentation hemodynamics, and clinically evident malperfusion) cannot be assessed when dissection occurs under general anesthesia and cardiopulmonary bypass, and the clinical context differs fundamentally from spontaneous ATAAD. Limitations include its retrospective, single-center design, the small sample size inherent to this rare complication, and unavoidable heterogeneity in reporting standards across published series, which precluded formal meta-analysis. The inclusion of two TAVR-associated intraoperative dissections also introduces a degree of procedural heterogeneity, which we acknowledge as a study limitation. Future multi-center registries with uniform definitions and core outcome sets are needed to refine risk stratification, directly compare surgical strategies in comparable anatomical scenarios, and prospectively validate parameters for cerebral and renal protection.

In summary, IAD management is both time- and strategy-sensitive. The relatively high mortality in our cohort likely reflects the complexity of cases and the scope of surgical reconstruction, but also reinforces the importance of rapid recognition, secure perfusion, appropriate repair, and meticulous organ protection. Standardized protocols and collaborative multi-center evidence will be critical to reducing the variability and lethality of this catastrophic complication.

## Figures and Tables

**Table 1:** Patient characteristics.

	*n*	%
Number of patients	31	
Female	15	48.4
Age (y)	65 ± 8.0	
BMI	25.0 ± 3.5	
BNP	132 (58.5, 405)	
EF (%)	60 (54, 63)	
Aortic sinus diameter (mm)	35 ± 5.0	
Ascending aortic diameter (mm)	40.9 ± 7.3	
SPAP	29 (27, 38)	
Bicuspid aortic valve	5	16.1
LVEF < 30%	2	6.5
NYHA III/IV	21	67.7
HTN	21	67.7
Atrial fibrillation	5	16.1
COPD	2	6.5
PVD	2	6.5
CRF	5	16.1
Preoperative dialysis	1	3.2
Neurological dysfunction	5	16.1
Diabetes mellitus	4	12.9
Previous cardiac surgery	7	22.6
Aortic aneurysm	3	9.7
Stable angina pectoris	12	38.7
Coronary malperfusion	15	48.4

Variables are presented as *n* (%), mean ± standard deviation, or median (IQR). IQR, interquartile range.

**Table 2:** Iatrogenic aortic dissection details.

	*n*	%
Index surgery		
CABG	4	12.9
AVR	3	9.7
MVR	3	9.7
MV (MIS)	7	22.6
Aortic surgery	2	6.5
Combination	10	32.3
MVR + TVR	4	12.9
CABG + VAR + LVR	2	6.5
CABG + AVR	1	3.2
CABG + MVR	1	3.2
CABG + VSD Repair	1	3.2
CABG + AAR	1	3.2
TAVR	2	6.5
Timing of Detection		
IO	28	90.3
POD 0	1	3.2
POD 1	1	3.2
POD 3	1	3.2
Mode of diagnosis		
Macroscopic	16	51.6
TEE	12	38.7
CT	1	3.2
Aortogram	1	3.2
Macroscopic+ TEE	1	3.2
Anatomical Entry Site		
Ascending aorta	25	80.6
Arterial cannulation entry	3	9.7
Clamp-associated entry	2	6.5
Descending aorta	1	3.2
Intraoperative Procedural Triggers		
Arterial cannulation	8	25.8
Cardioplegia cannulation	8	25.8
Aortic clamping	8	25.8
Other manipulation	3	9.7
Unknown	4	12.9

Variables are presented as *n* (%), mean ± standard deviation, or median (IQR).

**Table 3:** Operative details.

	*n*	%
Index Cannulation		
Femoral–femoral	3	9.7
Right atrium–femoral	8	25.8
Right atrium–aortic	3	9.7
Right atrium–axillary artery	1	3.2
Internal Jugular Vein–femoral	6	19.4
Vena Cava–femoral	1	3.2
Vena Cava–aortic	9	29.0
Operative extent		
Ascending aortoplasty	3	9.7
Aortic root/ascending aorta replacement	14	45.2
TEVAR	1	3.2
Aortic root/ascending aorta replacement+ Hemiarch replacement	7	22.6
Aortic root/ascending aorta replacement+ SUN’s surgery	4	12.9
Aortic root/ascending aorta replacement+ EVAR	1	3.2
Aortic root/ascending aorta replacement+ TEVAR	1	3.2
Intraoperative data		
Operative time (h)	10 ± 22.9	
Cross-clamp time (min)	165 ± 70.4	
Total circulatory arrest (n)	12	38.7
Total circulatory arrest (min)	28.7 ± 19.3	19.3
Lower body circulatory arrest (n)	6	19.4
Lower body circulatory arrest (min)	32.3 ± 9.7	9.7
CPB time (min)	342.6 ± 133.8	133.8
Minimum nasopharyngeal temperature (°C)	25.9 ± 3.0	3
Minimum rectal temperature (°C)	26.2 ± 5.5	5.5
Antegrade SCP		
Unilateral	8	25.8
Bilateral	4	12.9
RCP	1	3.2

Variables are presented as *n* (%), mean ± standard deviation, or median (IQR).

**Table 4:** Morbidity and mortality.

	*n*	%
Intraoperative RBC transfusion volume (u)	9.5 ± 5.3	
Hospital length of stay (days)	18 (14, 25)	
Postoperative mechanical ventilation time (h)	120.5 (39.5, 261.6)	
Return to OR/washout	9	29
ECMO	2	6.5
Outcome		
Neurological Complications	8	25.8
Stroke	7	22.6
SCI (permanent deficit)	1	3.2
Cardiovascular Complications	19	61.3
Low cardiac output	3	9.7
Heart failure	11	35.5
Atrial fibrillation	4	12.9
Ventricular fibrillation	1	3.2
Hepatic & Renal Dysfunction	19	61.3
Renal failure	14	45.2
Hepatic dysfunction	5	16.1
Respiratory Complications	18	58.1
Pneumonia	5	16.1
Type I respiratory failure	3	9.7
Type II respiratory failure	1	3.2
Pleural effusion	4	12.9
Tracheostomy	5	16.1
Hemorrhagic and Thrombotic Events	5	16.1
Re-exploration for bleeding	2	6.5
DVT	2	6.5
Arteriovenous fistula	1	3.2
Infectious Complications	4	12.9
Sepsis	2	6.5
GI complications	2	6.5
Mortality	12	38.7
In-hospital mortality	9	29.0
30-day postoperative mortality	3	9.7

Variables are presented as *n* (%), mean ± standard deviation, or median (IQR).

## Data Availability

The data presented in this study are available upon request from the corresponding authors.

## Abbreviations

The following abbreviations are used in this manuscript:

IAD	iatrogenic aortic dissection
BMI	body mass index
BNP	B-type natriuretic peptide
EF	ejection fraction
SPAP	systolic pulmonary artery pressure
LVEF	left ventricular ejection fraction
NYHA	New York Heart Association
HTN	hypertension
COPD	chronic obstructive pulmonary disease
PVD	peripheral vascular disease
CRF	chronic renal failure
CABG	coronary artery bypass grafting
AVR	aortic valve replacement or repair
MVR	mitral valve replacement or repair
MIS	minimally invasive surgery
TVR	tricuspid valve replacement or repair
VAR	ventricular aneurysm resection
LVR	left ventricular reconstruction
VSD	ventricular septal defect
AAR	ascending aortic replacement
TAVR	transcatheter aortic valve replacement
POD	postoperative day
TEE	transesophageal echocardiography
TEVAR	thoracic endovascular aortic repair
TEM	type–entry–malperfusion
EVAR	endovascular aortic repair
SUN’s surgery	stented elephant trunk procedure
CPB	cardiopulmonary bypass
SCP	selective cerebral perfusion
RCP	retrograde cerebral perfusion
RBC	red blood cells
OR	operating room
ECMO	extracorporeal membrane oxygenation
SCI	spinal cord injury
DVT	deep vein thrombosis
GI	gastrointestinal
NA	not available

## References

[1] Williams M.L., Sheng S., Gammie J.S., Rankin J.S., Smith P.K., Hughes G.C. (2010). Aortic dissection as a complication of cardiac surgery: Report from the Society of Thoracic Surgeons database. Ann. Thorac. Surg..

[2] Ram H., Dwarakanath S., Green A.E., Steyn J., Hessel E.A. (2021). Iatrogenic Aortic Dissection Associated With Cardiac Surgery: A Narrative Review. J. Cardiothorac. Vasc. Anesth..

[3] Singh A., Mehta Y. (2015). Intraoperative aortic dissection. Ann. Card. Anaesth..

[4] Jonker F.H., Schlosser F.J., Indes J.E., Sumpio B.E., Botta D.M., Moll F.L., Muhs B.E. (2010). Management of type A aortic dissections: A meta-analysis of the literature. Ann. Thorac. Surg..

[5] Tabry I.F., Costantini E.M. (2009). Acute aortic dissection early after off-pump coronary surgery: True frequency underestimated?. Tex. Heart Inst. J..

[6] Chavanon O., Carrier M., Cartier R., Hébert Y., Pellerin M., Pagé P., Perrault L.P. (2001). Increased incidence of acute ascending aortic dissection with off-pump aortocoronary bypass surgery?. Ann. Thorac. Surg..

[7] De Viti D., Dambruoso P., Izzo P., Dhojniku I., Raimondo P., Carbone C., Paparella D. (2021). Iatrogenic Acute Aortic Dissection in the Era of Minimally Invasive Cardiac Surgery—Experience of a Center and Review of Literature. Braz. J. Cardiovasc. Surg..

[8] Nappi F., Schoell T., Singh S.S.A., Salsano A., Abdou I., Gambardella I., Santini F.F., Fiore A., Garufi L., Demondion P. (2024). Aortic arch registry of type a aortic dissection (AoArch)—Rationale, design and definition criteria. J. Cardiothorac. Surg..

[9] von Aspern K., Leontyev S., Etz C.D., Haunschild J., Misfeld M., Borger M.A. (2022). Iatrogenic Type A Aortic Dissection: Challenges and Frontiers-Contemporary Single Center Data and Clinical Perspective. Aorta.

[10] Biancari F., Pettinari M., Mariscalco G., Mustonen C., Nappi F., Buech J., Hagl C., Fiore A., Touma J., Dell A.M. (2022). Outcome after Surgery for Iatrogenic Acute Type A Aortic Dissection. J. Clin. Med..

[11] Wang Y., Liu F., Song K., Lai H., Sun Y., Li J., Wang C., Ji Q. (2022). Immediate Recognition and Surgical Treatment of Iatrogenic Acute Type A Aortic Dissection Is Associated with Low Hospital Mortality and High Intermediate-Term Survival. Rev. Cardiovasc. Med..

[12] Ruchat P., Hurni M., Stumpe F., Fischer A.P., von Segesser L.K. (1998). Acute ascending aortic dissection complicating open heart surgery: Cerebral perfusion defines the outcome. Eur. J. Cardiothorac. Surg..

[13] Januzzi J.L., Sabatine M.S., Eagle K.A., Evangelista A., Bruckman D., Fattori R., Oh J.K., Moore A.G., Sechtem U., Llovet A. (2002). Iatrogenic aortic dissection. Am. J. Cardiol..

[14] Fleck T., Ehrlich M., Czerny M., Wolner E., Grabenwoger M., Grimm M. (2006). Intraoperative iatrogenic type A aortic dissection and perioperative outcome. Interdiscip. Cardiovasc. Thorac. Surg..

[15] Ketenci B., Enc Y., Ozay B., Gunay R., Cimen S., Gorur A., Tuygun A.K., Sargin M., Sari S., Demirtas M.M. (2008). Perioperative type I aortic dissection during conventional coronary artery bypass surgery: Risk factors and management. Heart Surg. Forum.

[16] Hurt A., Smith J.M., Engel A.M. (2008). Predictors and outcomes associated with intraoperative aortic dissection in cardiac surgery. J. Card. Surg..

[17] Lin T.Y., Chen Y.S., Chiu K.M., Hsu R.B., Yu H.Y., Wang M.J. (2009). Eight-year experience of intraoperative aortic dissection. Asian Cardiovasc. Thorac. Ann..

[18] Hwang H.Y., Jeong D.S., Kim K.H., Kim K.B., Ahn H. (2010). Iatrogenic type A aortic dissection during cardiac surgery. Interdiscip. Cardiovasc. Thorac. Surg..

[19] Leontyev S., Borger M.A., Legare J.F., Merk D., Hahn J., Seeburger J., Lehmann S., Mohr F.W. (2012). Iatrogenic type A aortic dissection during cardiac procedures: Early and late outcome in 48 patients. Eur. J. Cardiothorac. Surg..

[20] Rylski B., Hoffmann I., Beyersdorf F., Suedkamp M., Siepe M., Nitsch B., Blettner M., Borger M.A., Weigang E. (2013). Iatrogenic acute aortic dissection type A: Insight from the German Registry for Acute Aortic Dissection Type A (GERAADA). Eur. J. Cardiothorac. Surg..

[21] Narayan P., Angelini G.D., Bryan A.J. (2015). Iatrogenic intraoperative type A aortic dissection following cardiac surgery. Asian Cardiovasc. Thorac. Ann..

[22] Shea N.J., Polanco A.R., D’Angelo A., Bethancourt C.N., Sanchez J., George I., Patel V., Takayama H. (2019). Improving Outcomes of Iatrogenic Type A Aortic Dissection during Cardiac Surgery. Aorta.

[23] Harris K.M., Parikh N.B., Nienaber C.A., Woznicki E.M., Evangelista A., Schermerhorn M., Ouzounian M., Coselli J.S., Pai C.W., Ehrlich M.P. (2025). Iatrogenic aortic dissection: Insights from the International Registry of Acute Aortic Dissection. J. Thorac. Cardiovasc. Surg..

[24] Bauer S.J., Sugimura Y., Schoettler F.I., Immohr M.B., Suzuki T., Mehdiani A., Aubin H., Lichtenberg A., Akhyari P. (2025). Iatrogenic aortic dissection in minimally invasive cardiac surgery for atrioventricular valves and atrial structures. Eur. J. Cardiothorac. Surg..

[25] Liu T.X., Malaisrie S.C., Medina M., Whippo B., Baldridge A.S., Kruse J., Bryner B., Chiu S.F., Hodges K.E., Pham D.T. (2025). Surgical Outcomes of Iatrogenic Acute Type A Aortic Dissection During Catheter-Based Procedures: An STS Cardiac Surgery Database Analysis. Ann. Thorac. Surg..

